# Bone Pain in Multiple Myeloma (BPMM)—A Protocol for a Prospective, Longitudinal, Observational Study

**DOI:** 10.3390/cancers13071596

**Published:** 2021-03-30

**Authors:** Marta Diaz-delCastillo, Rebecca E. Andrews, Aritri Mandal, Thomas L. Andersen, Andrew D. Chantry, Anne-Marie Heegaard

**Affiliations:** 1 Department of Drug Design and Pharmacology, Faculty of Health and Medical Sciences, University of Copenhagen, 2100 Copenhagen, Denmark; amhe@sund.ku.dk; 2 Sheffield Myeloma Research Team, Department of Oncology and Metabolism, Mellanby Centre for Bone Research, University of Sheffield Medical School, Sheffield S10 2RX, UK; r.andrews@sheffield.ac.uk (R.E.A.); a.d.chantry@sheffield.ac.uk (A.D.C.); 3 Department of Haematology, Sheffield Teaching Hospitals NHS Foundation Trust, Royal Hallamshire Hospital, Sheffield S10 2JF, UK; aritri.mandal@nhs.net; 4 Department of Clinical Research and Department of Molecular Medicine, University of Southern Denmark, 5000 Odense, Denmark; Thomas.Levin.Andersen@rsyd.dk; 5 Department of Pathology, Odense University Hospital, 5000 Odense, Denmark; 6 Department of Forensic Medicine, University of Aarhus, 8200 Aarhus, Denmark

**Keywords:** multiple myeloma, pain, bone cancer, quality of life, bone innervation

## Abstract

**Simple Summary:**

Multiple myeloma is a bone marrow cancer that often causes bone pain, but little is known about the pain characteristics and mechanisms in this condition. This clinical study aims to: 1. characterize the type, location and intensity of pain in myeloma patients, and its effect of quality of life, and 2. investigate whether the nerve fibers in the bone of myeloma patients are altered. We will also explore whether pain intensity is correlated to blood indicators of inflammation or bone damage. Study results will help identify the mechanisms of myeloma-induced bone pain, allowing the development of new analgesics for these patients.

**Abstract:**

Multiple myeloma (MM) is a bone marrow neoplasia that causes bone pain in 70% patients. While preclinical models of MM have suggested that both nerve sprouting and nerve injury may be causative for the pain, there is a lack of clinical data. Thus, the primary aims of this clinical study are: (1) to provide a deep characterization of the subjective experience of pain and quality of life in MM patients; (2) to investigate disturbances in the bone innervation of MM patients. Secondary aims include exploring correlations between pain and serum inflammatory and bone turnover biomarkers. In a prospective, observational study (clinicaltrials.gov: NCT04273425), patients with suspected MM requiring a diagnostic iliac crest biopsy at Sheffield Teaching Hospital (UK) are invited to participate. Consenting patients answer seven standardized questionnaires assessing pain, quality of life and catastrophizing. Bone turnover biomarkers and inflammatory cytokines are measured in fasting serum samples, and bone innervation is evaluated in diagnostic biopsies. MM patients are invited to a follow-up upon completion of first line treatment. This will be the first deep characterization of pain in MM patients and its correlation with disturbances in bone innervation. Understanding how bone turnover and inflammation correlate to pain in MM is crucial to identify novel analgesic targets for this condition.

## 1. Introduction

Multiple myeloma (MM) is a hematological disorder in which terminally differentiated B-lymphocytes are abnormally cloned in the bone marrow, causing solitary plasmacytomas or spreading systemically to other bones. MM is usually associated with elevated serum or urine levels of paraprotein, while formal disease diagnosis requires the presence of ≥10% myeloma cells in the bone marrow and either end-organ damage (which can present in the form of hypercalcemia, renal insufficiency, anemia or osteolytic bone lesions) or a defined biomarker of malignancy [1]. Patients frequently develop myeloma bone disease as a consequence of increased osteoclast-mediated bone resorption and decreased osteoblastic bone formation, which often leads to the development of characteristic focal osteolytic lesions [2]. In most patients, MM is preceded by monoclonal gammopathy of undetermined significance (MGUS), which is characterized by elevated serum paraprotein and <10% myeloma cells in the bone marrow in the absence of end-organ damage [1]. MGUS affects 2%–5% of the population above 50 years of age, with a higher incidence in men, and it is estimated to evolve into MM in a stable rate of 1% per year [3].

While MGUS is considered an asymptomatic disorder, pain is a main cause of physician consultation in MM patients [4], and is more common in MM than in any other hematological cancer [5,6]. In a multi-center study collecting data from MM patients at any disease stage across 18 hematological clinics in the UK, 71% of myeloma patients reported pain (second most common symptom after fatigue, which affected up to 87%), which was described as “severe” or “overwhelming” by approximately 13% [7]. Pain is a feared symptom in cancer patients [8], that impairs their mood [9] and quality of life (QoL) [10]. Moreover, fear of pain has been found to predict up to 11% of the functional limitations of patients suffering from advanced cancer stages [8].

MM is commonly considered a disease of the elderly, as age is an important risk factor for its development [11]. Importantly, it has been shown that QoL is one of the primary concerns in elderly cancer patients, who often find it more important to improve their QoL than their overall survival [12]. Therefore, incorporating measures of pain and QoL in studies assessing clinical interventions for the treatment of MM is of utmost importance. Pain in MM can be grossly classified as either bone pain, arising as a consequence of disease progression; chemotherapy-induced neuropathic pain, as a side effect of the main anti-myeloma medications commonly used to treat the disease, or procedural pain, derived from the invasive medical procedures conducted to treat the myeloma. However, despite the growing focus on pain management, knowledge on the characteristics and baseline presentation of pain and QoL in myeloma patients is scarce. Moreover, the underlying mechanisms of myeloma-induced bone pain are grossly understudied. In preclinical MM models displaying pain-related behaviors, both bone denervation [13] and nerve sprouting [14] of the bone marrow have been described; however, it remains unknown whether this reflects the clinical landscape. Therefore, clinical studies addressing changes in bone innervation in MM patients and their correlation to pain may shed some light on the mechanisms of myeloma-induced bone pain, and bone cancer pain in general.

As novel therapies are being developed, a growing subset of patients live with myeloma as a long-lasting disease, alternating between phases of clinical remissions and relapses. Thus, understanding and improving the pain and subsequent QoL of myeloma patients has become increasingly important. The aim of this study is to provide a deep characterization of the perception of pain and QoL in firstly diagnosed MM patients. Because pain is a subjective, multidimensional experience that comprises sensory, emotional and cognitive dimensions, this study aims to collect patient information through a constellation of standardized, validated questionnaires; data from MM patients will be compared with that from patients receiving a negative myeloma diagnosis (presumably MGUS patients). To further understand the mechanisms of myeloma-induced bone pain, bone innervation and serum inflammatory and bone turnover biomarkers will be evaluated in MM patients and compared with patients with a negative myeloma diagnosis. Moreover, data from MM patients at diagnosis will be compared with data collected upon completion of first-line treatment, and correlations between pain, bone innervation and serum biomarkers will be investigated.

## 2. Research Methods and Analyses

### 2.1. Study Design and Setting

**Figure 1:**
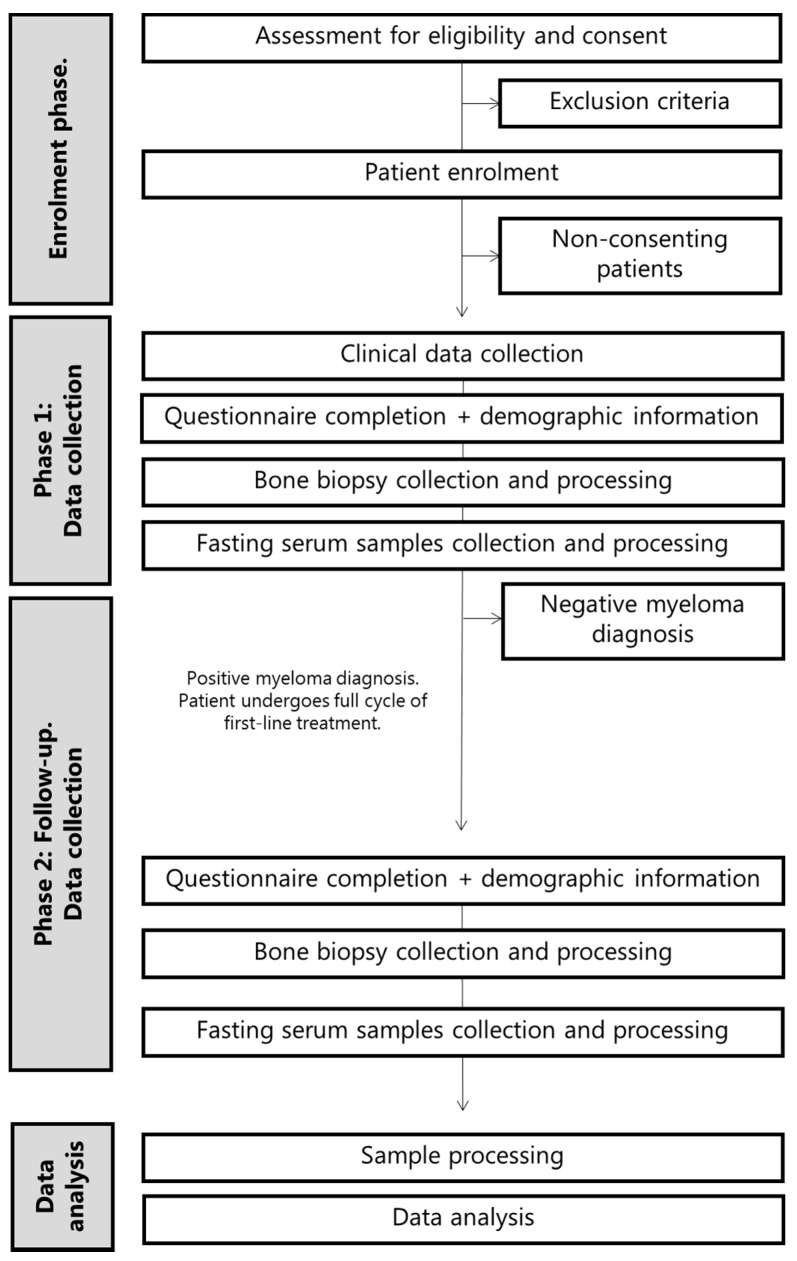
Flow diagram indicating the study design.

A prospective, observational study in a cohort of patients undergoing a diagnostic bone marrow biopsy for suspected MM. This is a single-center study within an international collaboration; patients will be recruited and samples obtained at Sheffield Teaching Hospital NHS Foundation Trust, Sheffield, UK. Sample analyses will be performed at University of Copenhagen, Copenhagen, Denmark, or University of Southern Denmark, Odense, Denmark. The Danish Association for Multiple Myeloma was contacted during conceptualization; they confirmed the study’s relevance to MM patients. A flow diagram of the study design can be found in Figure 1.

The primary study aims are:

- To provide a deep characterization of the subjective experience of pain (including pain type, location and severity) and QoL in MM patients at diagnosis, compared to patients with a negative myeloma diagnosis (control group, presumably MGUS patients).
- To evaluate disturbances in bone innervation (presence, location and density of nerve fibers innervating the bone) in MM patients at diagnosis, compared to patients with a negative myeloma diagnosis.

Secondary aims of this study include evaluating the levels of bone turnover biomarkers and serum inflammatory cytokines in MM patients at diagnosis, compared to patients with a negative myeloma diagnosis.

Additionally, data on pain parameters and QoL, bone innervation and serum biomarkers from MM patients at diagnosis will be compared with data collected following first-line treatment. Correlations between pain and disturbances in bone innervation and serum biomarkers in MM patients before and after treatment will also be investigated.

### 2.2. Study Population

**Figure 2:**
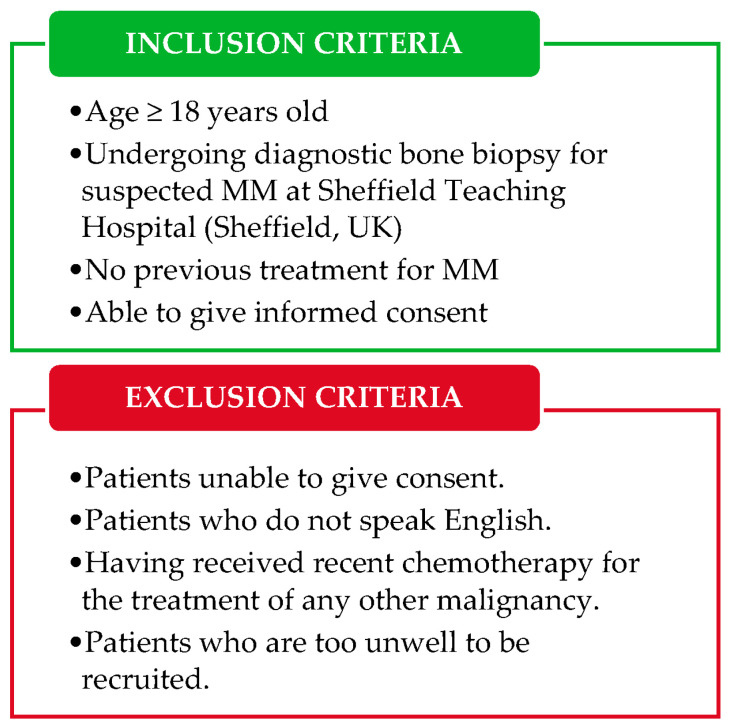
Summary of the patient’s eligibility criteria for the study. MM = multiple myeloma.

All patients undergoing a diagnostic bone marrow biopsy for suspected MM between October 2019 and June 2021 will be invited to participate by their Hematology Consultant during their scheduled medical appointment. Inclusion and exclusion criteria are specified in Figure 2. Due to the impact of the COVID-19 pandemic on healthcare institutions, the study was paused between March 2020 and August 2020; thus, patient recruitment is expected to continue until June, 2021. The study population will comprise all consenting patients undergoing a diagnostic bone biopsy for suspected MM at Sheffield Teaching Hospital; data from patients with a negative MM diagnosis (confirmed MGUS or smoldering multiple myeloma, SMM, patients) will be used as control. Enrolled patients receiving a positive MM diagnosis will be invited to participate in a study follow-up, upon completion of first-line treatment (approximately 8 months after diagnosis). Patients may withdraw from the study at any point; collected data and tissue will be retained.

### 2.3. Study Objectives

1. To provide an in-depth characterization of the subjective experience of pain and QoL in MM patients through the completion of standardized questionnaires regarding general pain, bone pain, neuropathic pain, QoL and catastrophizing. Questionnaires from MM patients will be compared with those of patients receiving a negative myeloma diagnosis (control group).
2. To evaluate disturbances in the presence, location and density of sensory and sympathetic nerve fibers innervating the bone marrow of myeloma patients. Biopsies from MM patients will be compared with those of patients receiving a negative myeloma diagnosis (control group).
3. To evaluate changes in the levels of serum biomarkers of bone turnover (carboxy-terminal collagen crosslinks-1, CTX-1, and pro-collagen type 1 N-terminal pro-peptide, P1NP) in MM patients. Levels of bone turnover biomarkers in the serum of MM patients will be compared with those of patients receiving a negative myeloma diagnosis (control group).
4. To evaluate the levels of inflammatory serum cytokines and chemokines in MM patients. Levels of inflammatory biomarkers in the serum of MM patients will be compared with those of patients receiving a negative myeloma diagnosis (control group).
5. To compare data on pain and QoL, bone innervation and serum biomarkers of bone turnover and inflammation in MM patients at diagnosis and following first-line treatment.
6. To evaluate correlations between the self-reported experience of pain and disturbances in bone innervation in MM patients at diagnosis and following first-line treatment.
7. To evaluate correlations between the self-reported experience of pain and serum levels of bone turnover biomarkers in MM patients at diagnosis and following first-line treatment.
8. To evaluate correlations between the self-reported experience of pain and serum levels of inflammatory cytokines in MM patients at diagnosis and following first-line treatment.

### 2.4. Study Procedures

#### 2.4.1. Objectives 1 and 5: In-Depth Characterization of Pain in MM Patients

Patients who consent to participate will receive a booklet with a total of seven standardized questionnaires (Table 1), which will provide a deep characterization of their subjective experience of pain. Data from myeloma patients at diagnosis will be compared with that of patients receiving a negative myeloma diagnosis (control group). MM patients will be asked to complete the same set of questionnaires upon completion of first-line treatment; data from MM patients at diagnosis and following first-line treatment will be compared. The questionnaires include:

1. BPI (Brief Pain Inventory, short version): two-factor questionnaire assessing the severity and interference of pain [15,16]. The severity component assesses location and severity of the pain, as well the analgesic relief provided by current therapy, if any. The interference component is composed by an affective and an activity subdimension. The BPI includes a Visual Analogue Scale (VAS) for assessment of pain intensity, which will be used to establish correlations between pain and disturbances in bone innervation, serum inflammatory and serum bone turnover biomarkers. Data from this questionnaire will allow to draw comparisons between pain severity and interference between MM patients and controls.
2. FACT–BP (Functional Assessment of Cancer Therapy–Bone Pain): Initially developed for the assessment of pain in bone metastases, it measures bone pain and its effect on quality of life. This will provide information on whether myeloma patients suffer from bone pain, compared to controls. Moreover, this questionnaire is sensitive to clinical changes following therapy [17], and will provide important information on whether first-line anti-myeloma treatment improves bone pain and its effect on quality of life in myeloma patients.
3. PCS (Pain Catastrophizing Scale): assessment of the extent of catastrophizing, which is subdivided in three domains: rumination, magnification and helplessness [18]. Mounting evidence suggests that catastrophizing (i.e., enhancing negative, anxiety-inducing thoughts) plays an important role in the subjective experience of pain; to our knowledge, this will be the first data on the role of catastrophizing in myeloma-induced bone pain.
4. EORTC QLQ-C30 (European Organization for the Research and Treatment of Cancer Quality of Life Questionnaire-Core 30): assessment of quality of life in cancer patients. This questionnaire contains a measure of global health status, functional scales (including physical, role, emotional, cognitive and social functioning) and symptoms scales (including fatigue, nausea/vomiting, pain, dyspnea, insomnia, appetite loss, constipation, diarrhea and financial difficulties) [19]. As this questionnaire is widely used to assess the effect of cancer on QoL, it will allow to contextualize the effect of myeloma on QoL in comparison to other cancer types.
5. EORTC MY20 (European Organization for the Research and Treatment of Cancer Quality of Life Questionnaire-myeloma module 20): Supplementary module to administer alongside the EORTC QLQ-C30 for the assessment of disease symptoms, side effects of treatment, future perspective and impact of disease on body image perception [20]. This questionnaire will allow comparisons to other cohorts of myeloma patients.
6. EORTC CIPN (European Organization for the Research and Treatment of Cancer Quality of Life Questionnaire-chemotherapy-induced peripheral neuropathy module): assessment CIPN through a sensory, a motor and an autonomic scale [21]. Administration of this questionnaire at diagnosis will provide baseline data to compare the follow-up data to, allowing the identification of CIPN development after treatment.
7. painDETECT: identification of a possible neuropathic component of pain [22]. This questionnaire, in combination with the EORTC CIPN, will allow the distinction between myeloma-induced neuropathy and chemotherapy-induced peripheral neuropathy”.

Additionally, patients will be asked to fill in a customized demographic questionnaire regarding their marital status, whether they cohabitate with their children, their postal code, country of origin, level of education and employment status. Patients will also be asked to answer the question: “is there any important information regarding your pain experience or quality of life that you feel has not been covered in the given questionnaires?”

Patients will receive the questionnaire booklet during their routine hospital visits, and will be asked to return it in person or mail it back to Sheffield Teaching Hospital, U.K. If a patient consents to participate in the study but feels too unwell to answer the questionnaires on her/his own, a structured interview, in which a researcher asks the questions and notes down the answers, will be offered. Record of self-assessed questionnaires vs. structured interviews will be kept.

#### 2.4.2. Objectives 2 and 5: Immunohistological Characterization of Nerve Fibers Innervating the Bone Marrow in MM

Current guidelines for the diagnosis of MM require the presence of at least 10% clonal cells infiltrating the bone marrow [1]. Therefore, to effectively diagnose MM, the healthcare team routinely extracts diagnostic trephine bone biopsies of the iliac crest and process them for quantification of MM cell infiltration.

Upon finalizing the standard pathology procedures, the research team will access the bone biopsies (both diagnostic and following first-line treatment) and perform an immunohistological characterization of the existence, location and density of neuronal fibers. Bone biopsies from MM patients will be compared to those from patients with a negative myeloma diagnosis. Serial sections will be subjected to multiplex-immunostainings for the pan-neuronal marker PGP9.5 (protein gene product 9.5) combined with the sympathetic nerve fiber marker TH (tyrosine hydroxylase), allowing the distinction between sympathetic and non-sympathetic (presumably sensory) fibers, and the myelinated axon marker NF200 (neurofilament 200), allowing the distinction between myelinated and non-myelinated fibers. A similar protocol for nerve characterization in human bone biopsies has previously been optimized in-house [23]. The analysis will estimate the number of nerve profiles per marrow area (mm^2^) of all PGP9.5-positive nerve profiles and of the nerve fibers’ subtypes, their association with a nerve cluster [23] and the percentage of nerve profiles with another nerve profile within 25 µm. Moreover, the multiplex-immunostaining will be combined with markers for osteoclasts (tartrate-resistant acid phosphatase 5b, TRAcP5b), blood vessels (CD34/CD31) and myeloma cells (CD138), allowing the analysis of the neuronal fibers proximity to blood vessels, myeloma cells and osteoclasts. The analysis will estimate the percentage of nerve profiles with an osteoclast, blood vessel, myeloma cell and bone within 25 µm.

Following first-line treatment, it is standard of care to extract a new bone biopsy to evaluate the presence of malignant plasma cells and assess treatment efficacy. Follow-up biopsies from enrolled MM patients will be analyzed to address the differences in bone innervation at diagnosis and upon completion of first-line treatment. Analyses will be performed by a researcher blinded to patient’s disease diagnosis.

#### 2.4.3. Objectives 3, 4 and 5: Quantification of Serum Biomarkers of Bone Turnover and Inflammation

To assess the levels of bone turnover or inflammatory biomarkers in MM patients and patients receiving a negative myeloma diagnosis, fasting serum samples will be collected. Bone resorption and formation biomarkers (CTX-1 and P1NP, respectively) will be quantified by enzyme-linked immunosorbent assay. Serum inflammatory chemokines and cytokines will be quantified using a multiplex array system; measurements will include (but not be limited to) chemokine (C-C motif) ligand 3 (CCL3), epidermal growth factor (EGF), fibroblast growth factor (FGF-2), granulocyte colony-stimulating factor (G-CSF), Granulocyte-macrophage colony-stimulating factor (GM-CSF), interferon α2 and γ, interleukins 1α, 1β, 2, 3,4,5,6,7,8,9,10,13, 15, 18, platelet derived growth factor (PDGF), transforming growth factor (TGF)α, and tumor necrosis factor (TNF) α and β. Fasting serum samples from MM patients will also be collected following first-line treatment; changes in bone turnover and inflammatory biomarkers in MM patients upon completion of first-line treatment will be evaluated.

#### 2.4.4. Objectives 6, 7 and 8: Correlation Analyses

Data on bone innervation and serum bone turnover and inflammatory biomarkers in MM patients will be correlated to self-reported measures of pain, both at diagnosis and following first-line treatment.

#### 2.4.5. Sample Size Calculation

Power calculations for this study have been performed in relation to pain intensity scores between firstly diagnosed, untreated, MM patients and patients receiving a negative myeloma diagnosis (control group). The power calculation has been performed on the primary aim: pain in multiple myeloma, as measured in the numeric rating scale contained in the BPI, in which worse pain is scored in the 11-point numeric scale (0 = no pain, 10 = worst imaginable pain). Power calculations were performed using a pain intensity difference and a standard deviation of 2.75 in the 11-point numeric scale, as previously reported for MM patients [24]. For a power of 80%, our study requires a sample size of 17 patients per group.

The recruitment phase of this study will take place from October 2019 to June 2021. Based on historical experience, it is expected that 50 eligible study candidates will be identified and at least 35 are expected to participate, as the study involves minimal invasive procedures. We expect that at least half of the enrolled patients will be diagnosed with multiple myeloma, and data from patients with a negative myeloma diagnosis (MGUS or SMM patients) will be used as control.

## 3. Discussion

Pain is one of the most prevalent and feared cancer symptoms, and, together with fatigue, the main complain of MM patients [4,7]. The aim of this study is to provide a deep characterization of the subjective experience of pain, as perceived by MM patients, at time of diagnosis. Moreover, we will analyze the effect of first-line treatment on both pain and QoL.

Over the last few decades, methods to monitor symptoms and adverse effects, which ultimately contribute to the patients’ QoL, have gained attention. Traditionally, adverse effects are monitored by the clinicians following the Common Terminology Criteria for Adverse Events guidelines [25]. However, recent studies have shown that patient-reported outcomes (PRO) may be more accurate measures of patients´ symptoms, as clinicians often underestimate adverse effects [26]. This is also true in MM, as highlighted by a recent Danish study in which a poor correlation between PRO and clinician-reported adverse effects was found for all evaluated symptoms, including peripheral neuropathy, diarrhea, nausea and vomiting, fatigue, insomnia and appetite loss [27]. To overcome this, our study will apply a constellation of widely used, validated PROs to characterize myeloma-induced bone pain. These questionnaires include the short version of the BPI, which is the most commonly used tool for the evaluation of cancer pain [28] and assesses least, worst and average pain through numeric rating scales, which have been proved to have more power to detect differences in pain intensity than verbal categorical rating scales [29]. Additionally, the BPI evaluates the affective component of pain by assessing the relations with others, life enjoyment and mood, and the interference of pain with daily life, including walking, general activity and work. In combination with the FACT-BP, specifically developed for the assessment of bone pain, and the PCS, which focuses on catastrophizing and fear of pain, we expect to be able to capture relevant information on all pain dimensions (sensory, cognitive and affective) in myeloma patients.

To correlate the pain results to QoL, recruited patients will fill out the EORTC QLQ-C30 and EORTC QLQ-MY20, which are commonly used questionnaires for the evaluation of QoL in cancer and myeloma patients, respectively. As both these questionnaires have commonly been used in clinical studies with MM patients, they will allow comparisons to the published data regarding QoL in other patient cohorts [30,31,32].

Due to the preclinical evidence of nerve disturbances in the bones of myeloma-bearing mice presenting pain-like behaviors [13,14], we suspect that bone pain in myeloma patients may have a neuropathic component, even before receiving medication that may cause neuropathy (e.g., chemotherapy, proteasome inhibitors). For that reason, the EORTC QLQ-CIPN20 and painDETECT questionnaires will be administered, in an attempt to identify whether the pain of non-chemotherapy-treated myeloma patients (baseline measurements) presents a neuropathic component. We expect that the combination of these validated, standardized questionnaires will provide a deep characterization of pain in this patient population, allowing comparisons with other well-characterized populations such as patients with metastatic bone disease.

A common limitation of PRO to monitor HRQoL in longitudinal studies is the lack of compliance, which can compromise the power of clinical studies [33] and cofound the results (i.e., if the frailest patients do not complete PROs and drop out, the study results may overestimate QoL and underestimate toxicity and symptom burden). Recently, a Danish multicenter study evaluated, for the first time, a range of strategies to increase compliance to PRO in a population of myeloma patients [34]. In this study, myeloma patients at all disease stages were invited to participate by filling in PROs at 12 months intervals during a total of 24 months; the only exclusion criteria were set up as patients who do not speak Danish, or patients with a diagnosed psychiatric condition, so that the “real population” of myeloma patients in Denmark was represented. The results of this study showed that real-time monitoring of non-response patients, sending electronic reminders and educating study nurses leads to an increase of up to 95% PRO response rate, with frailty being a strong predictor for non-completion of self-administered questionnaires [34]. In an effort to improve compliance, patients recruited for this study will be given the option to fill in the questionnaire booklet themselves (self-reported PRO) while waiting for procedures in the hospital or take them home and return them in their next appointed visit. Follow-up calls will be placed to patients who fail to return the questionnaires within the agreed time-period. Thus, we expect compliance in this study to be high.

In the clinic, diagnosis of MM requires the immunohistological characterization of clonal plasma cells in the bone marrow. In this study, we will access diagnostic iliac crest biopsies. Sensory and sympathetic nerve profiles will be evaluated in serial sections, allowing the study of correlations between disturbances in bone innervation and pain parameters. We have previously observed tumor-induced nerve injury in a mouse model of myeloma-induced bone pain, which we hypothesize may be causative for the pain [13], while nerve sprouting has also been described in animal models of myeloma [14]. However, due to the lack of human studies on bone innervation in myeloma patients, it is unknown whether this translates to the clinical situation. Previous studies of bone innervation in primary hyperparathyroidism patients revealed that the majority of nerve profiles are closely associated with blood vessels, and that the density of nerve profiles innervating the bone marrow is higher above bone remodeling surfaces [23]. Moreover, histological studies of the bones of MM patients have shown that myeloma cells often disrupt the canopies covering bone remodeling compartments, leading to the fusion of myeloma cells with osteoclasts to create osteoclast hybrid cells [35]. To the best of our knowledge, no study has characterized nerve fibers in the bone of MM patients. Thus, immunohistological characterization of osteoclasts and clonal plasma cells in relation to nerve fibers and blood vessels in these patients will contribute towards elucidating the peripheral mechanisms of myeloma-induced bone pain.

As MM is characterized by the uncoupling of bone resorption and formation and the consequent development of osteolytic bone lesions, we will quantify bone turnover through analyzing the systemic levels of CTX-1 and P1NP. Presently, it remains unclear whether osteolytic lesion development is a direct driver of bone pain, as bisphosphonate treatment has shown dramatic effects on bone remodeling but their analgesic effect on bone cancer remains controversial [36]. To our knowledge, this study will be the first to evaluate correlations between bone resorption biomarkers and pain in MM. Additionally, we will investigate correlations between circulating cytokine levels and pain. It has previously been shown that interleukin-6 is positively correlated with pain intensity in advanced, stable multiple myeloma [37] and several other inflammatory cytokines that have been correlated to pain in patients with cancer-induced bone pain [38,39]. This will be the first study to investigate correlations between circulating cytokines and pain in firstly-diagnosed myeloma patients.

### Limitations of the Study

To date, data characterizing pain and QoL from the “real” MM population (patients who are not included in clinical studies are often older and frailer) or across different disease stages (i.e., before diagnosis, after first-line treatment) is scarce [32]. A possible bias of this study is that the older and frailer patients may not consent to participate; to reduce this bias, structured interviews will be offered to patients who feel too unwell to complete the study questionnaires on their own. Another possible bias is that, in this study, only English-speaking patients are asked to participate, as resources did not allow for translation of study documents to incorporate non-English speaking participants to give informed consent.

A major limitation of this study is the lack of appropriate bone biopsy controls. In this study, all patients undergoing diagnostic bone biopsies for suspected MM are invited to participate, thus including a subpopulation of confirmed MM patients and another with a negative diagnosis (confirmed MGUS or SMM patients, who will often present with elevated serum paraprotein but less than 10% malignant plasma cells in bone), who will serve as negative controls. However, it should be noted that biopsies from MGUS patients may also display bone or nerve disturbances, as they may also present low levels of abnormal clonal cells in the bone marrow. Obtaining bone biopsies from healthy volunteers could be considered, but due to the high invasiveness of the procedure, which often causes pain and discomfort for the patient, it was considered unattainable.

Another important limitation of the study is the area from which the biopsy will be extracted. Diagnostic trephine bone biopsies are generally extracted from the iliac crest, while MM patients may present bone pain, nerve disturbances or plasmacytomas in any other body area. Obtaining a pain-guided bone biopsy (i.e., a biopsy from the specific bone in which a patient describes a painful sensation) could be considered. However, all patients undergoing diagnostic bone biopsies at Sheffield Teaching Hospital will be included in this study, whether they present with bone pain or not, in an attempt to characterize pain in the entire MM population. Furthermore, collecting extra bone biopsies solely for research purposes would expectably decrease the number of consenting patients in the study. For that reason, only diagnostic biopsies will be evaluated and systemic bone innervation changes are expected to be observed. As hip is a common site of bone pain in MM [39], we expect to be able to identify nerve disturbances in the trephine bone biopsies collected for this study.

## 4. Ethics and Dissemination

This study is conducted in agreement with the Declaration of Helsinki and local laws and regulations. Eligible patients are informed about the study and given enough time to consider whether they want to participate. Written information is provided to all patients who may be interested in participating. Written informed consent is obtained by the research team.

Data will be analyzed with the appropriate statistical software and will be reported in compliance with the Strengthening the Reporting of Observational studies in Epidemiology (STROBE) guidelines [40], while inclusion of relevant items in the study protocol has been ensured following the Standard Protocol Items: Recommendations for Interventional Trials (SPIRIT) checklist [41] (Supplementary File S1).

Personal data will be handled confidentially. Patients will be initially approached by members of their standard health care team, and only after receiving explicit consent, research staff will approach them and invite them to participate in the study. Study participants will be assigned a unique personal study code. All research data, including the physical questionnaires’ booklets administered to the patients, will be pseudo-anonymized to this personal code. An Excel spreadsheet where the study code is linked to the patient´s hospital code (i.e., the decryption key) will be stored in a password-protected National Health Service computer and backed up on a secure, encrypted National Health Service share drive. All physical files containing personal data, such as consent sheets, will be stored in a secure location in a locked office, in a building protected by CCTV cameras and security guards. No identifiable data will be exported outside the United Kingdom, and patients will be explicitly consented to allow access to their personal data to research staff outside the medical team that is directly implicated in their standard of care.

The results of this study will be disseminated by publication in peer-reviewed scientific journals and participation in scientific conferences; authorship will be based upon the Vancouver recommendations. A layman summary of the study results will be administered to the study participants who consent to it.

## Figures and Tables

**Table 1:** Patients recruited in the Bone Pain in Multiple Myeloma Study are provided with a booklet containing seven validated, standardized questionnaires assessing pain, quality of life and catastrophizing. MM = Multiple Myeloma.

	Full Questionnaire Name	Abbreviated Name	Copyright	Objective	Number of Items
PAIN	Short Brief Pain Inventory [15,16]	BPI	Dr. Charles S. Cleeland, MD Anderson Centre	Pain severity and interference (affective and activity subdimensions)	9
	Functional Assessment of Cancer Therapy-Bone Pain [17]	FACT-BP	Dr. David Cella, FACIT system	Quality of Life in patients with bone pain	16
	Pain Catastrophizing Scale [18]	PCS	Dr. Michael JL Sullivan	Measure of the extent to which a patient catastrophizes pain	13
QUALITY OF LIFE	European Organization for the Research and Treatment of Cancer: Quality of Life Questionnaire-Core 30 [19]	EORTC QLQ-C30	European Organization for the Research and Treatment of Cancer	Quality of Life in cancer patients	30
	European Organization for the Research and Treatment of Cancer: Quality of Life Questionnaire-myeloma module 20 [20]	EORTC QLQ-MY20	European Organization for the Research and Treatment of Cancer	Quality of Life in MM patients	20
NEUROPATHY	European Organization for the Research and Treatment of Cancer Quality of Life Questionnaire-chemotherapy-induced peripheral neuropathy module 20 [21]	EORTC QLQ-CIPN20	European Organization for the Research and Treatment of Cancer	Characterization of chemotherapy-induced peripheral neuropathy	20
	painDETECT [22]	painDETECT	Pfizer Pharma GmbH, Germany	Identification of a neuropathic pain component	11

## Data Availability

No new data were created or analyzed in this study. Data sharing is not applicable to this article.

## Supplementary Materials

The following are available online at https://www.mdpi.com/article/10.3390/cancers13071596/s1, File S1: SPIRIT 2013 Checklist.
